# Supplementary material for: vwa1 Knockout in Zebrafish Causes Abnormal Craniofacial Chondrogenesis by Regulating FGF Pathway
Source: Genes (Basel). 2023 Mar 30;14(4):838. doi: 10.3390/genes14040838 (PMC10137681; doi:10.3390/genes14040838)
Supplement: Supplementary file 1 [file genes-14-00838-s001.zip › genes-2276560-supplementary.pdf]

Supplemental Table S1. gRNA targets and verification primers used to amplify the target region.

Primer name	Sequence
vwa1 gRNA Target1 F	TAATACGACTCACTATAGGCTCACATGATCTGGGCCCGTTTTAGAGCTAGAAATAGC
Target1 verification primer-F	GTTGAATTGCTGTGAAGGCGA
Target1 verification primer-R	CAGTAGGCGGACTCACGAC
vwa1 gRNA Target2 F	TAATACGACTCACTATAGGTCGTGAGTCCGCCTACTGGTTTTAGAGCTAGAAATAGC
Target2 verification primer-F	TACGAGTTCTCCCGCATGGT
Target2 verification primer-R	ACCCACCAATGATTGAGTTCC
vwa1 gRNA Target3 F	TAATACGACTCACTATAGGGATCTGTCCCGTCCTCATGTTTTAGAGCTAGAAATAGC
Target3 verification primer-F	CATCCGCGCTGAGAGACTG
Target3 verification primer-R	TGTGTATGTGCTACTCACCTGT
vwa1 gRNA Target4 F	TAATACGACTCACTATAGGCACTCGAAGCGGGCCACTGTTTTAGAGCTAGAAATAGC
Target4 verification primer-F	CAGATCCAGCTGTTGTCCTCTT
Target4 verification primer-R	GTGTATTAATGTGAACTCTCCTCAC
vwa1 gRNA R	AAAAAAAGCACCGACTCGGTGCCAC

Supplemental Table S2. Primers used in RT-qPCR of *vwa1*, *fgfr1a*, *fgfr2*, *fgfr3*, *fgfr4*, *fgf8a*, *fgf8b*, and *runx2a*.

Primer name	Sequence
vwa1 qPCR F	CATCATCGGCGAAGACCTGA
vwa1 qPCR R	GATCTGTCCCGTCCTCATGG
fgfr1a qPCR F	CGGAAACTCTATCGGCCACT
fgfr1a qPCR R	GGAGTCCACAGACACTGTTACC

fgfr2 qPCR F	ATAGGTGTGTTTCTGATCGCCT
fgfr2 qPCR R	TGACGACACTGTTACCTGGC
fgfr3 qPCR F	AGTAGTTCTGTCCTGCACGC
fgfr3 qPCR R	CCTGCCTCGTCCTCATCTTC
fgfr4 qPCR F	CTCTTGGACGTGTTGGAACG
fgfr4 qPCR R	GGAATGCCATCAGGCCCATA
fgf8a qPCR F	GCCGTAGACTAATCCGGACC
fgf8a qPCR R	TGGCTTCACTCTTCAACGCT
fgf8b qPCR F	TGAGGCTGAAATCATCGAGGT
fgf8b qPCR R	GTTTACCGCTGGTTCGGCTA
runx2a qPCR F	CAACTTTCTGTGCTCGGTGC
runx2a qPCR R	GCGGTGGGTTCGTGAATACT
actb1 qPCR F	ACCACGGCCGAAAGAGAAAT
actb1 qPCR R	ATGTCCACGTCGCACTTCAT

Supplemental Table S3**.** Primers used in the cloning of *crestin*, *dlx2a*, *tbx1*, *barx1*, *nkx2.3*, *fgf8a*, *fgfr2*, *fgfr3*, *sox9a*, and *col2a1a* for RNA probe synthesis before whole-mount in situ hybridization.

**Primer name**	**Sequence**
crestin in situ F	GAGAAGCCCTCATCAGAGAGTTTG
crestin in situ R	GTTGCTTGTCAGGCAGAATCAGG
dlx2a in situ F	CACAGTTCTGCTTTGCGTCG
dlx2a in situ R	CCCAAGTCGGCAGAGTCAAA
tbx1 in situ F	GCAGCTGTCCCATTTTTGCG
tbx1 in situ R	ACGGCGGTAAATCTGGTCTC
barx1 in situ F	CTGGGCGGATCAGACTTCTC
barx1 in situ R	GCTTCTCGTGTCCTCTCCTG
nkx2.3 in situ F	TCGTGTTTTCTCGGAGGTGG
nkx2.3 in situ R	GCGCATTAGTGGACGTGTTC
sox9a in situ F	CCTCGACCCCTACCTGAAGA
sox9a in situ R	GGCGGGAGGTATTGGTCAAA
col2a1a in situ F	TCTGAAGTCCATCAACGGGC
col2a1a in situ R	TTTTCCGTCACGCTAAACGC

fgf8a in situ F	GCCGTAGACTAATCCGGACC
fgf8a in situ R	TGGCTTCACTCTTCAACGCT
fgfr2 in situ F	GACCCTGATCATGGAGAGCG
fgfr2 in situ *R*	TGACGACACTGTTACCTGGC
fgfr3 in situ F	CGCAAGACTTCCTCCTTCCC
fgfr3 in situ *R*	AGTGTGCTTGATTGTCCCGT

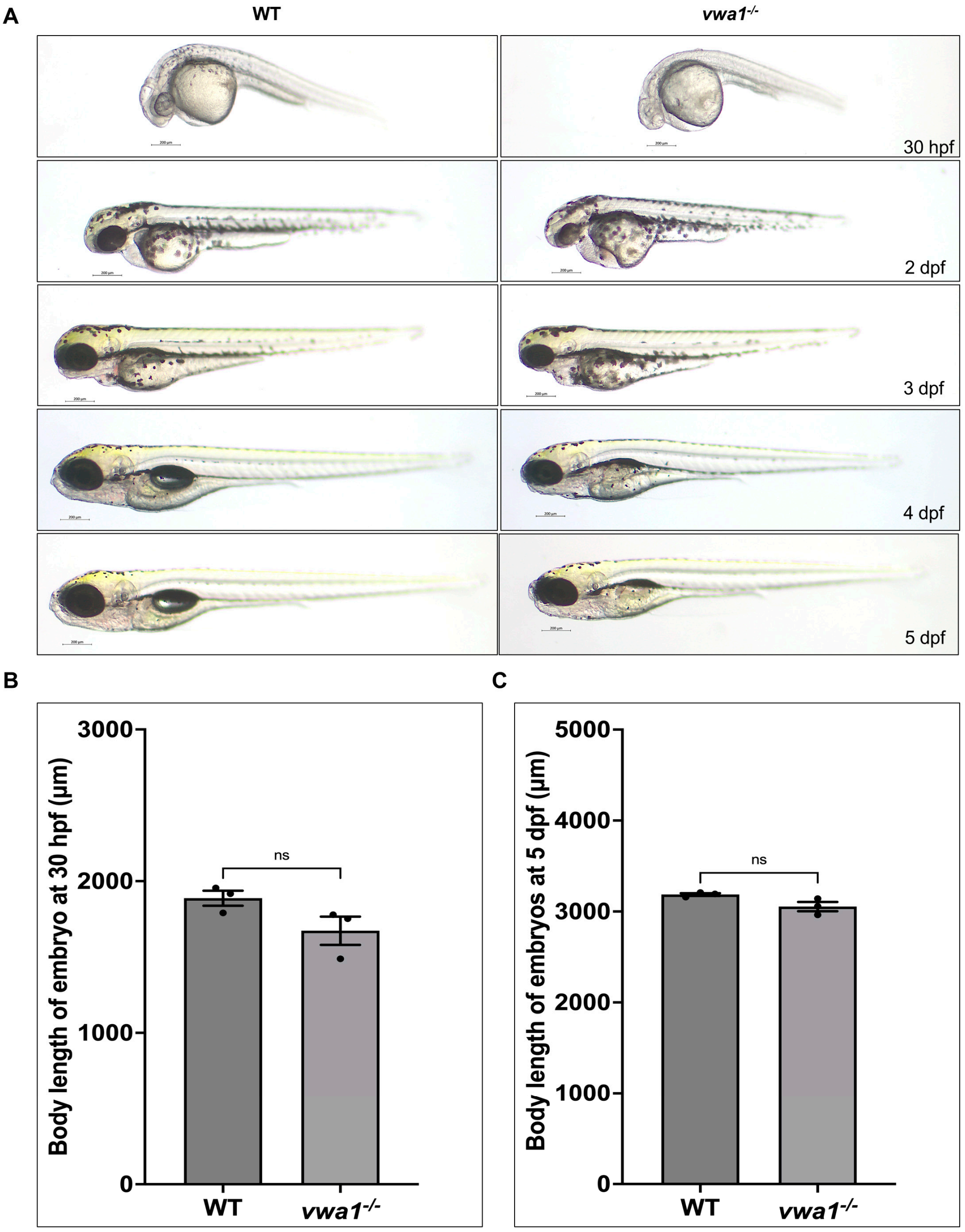

Supplemental Figure S1. Comparison of the body sizes of *vwa1*$^{-/-}$ mutants and wildtype controls from 30 hpf to 5 dpf. (**A**) Light microscopy demonstrated some *vwa1*$^{-/-}$ mutants had shorter body lengths and smaller head circumferences than the wildtype control (WT). (**B**, **C**) There was no statistical size difference in body length

between mutants and WT at 30 hpf and 5 dpf, although the average body length of the mutants was slightly shorter than WT.

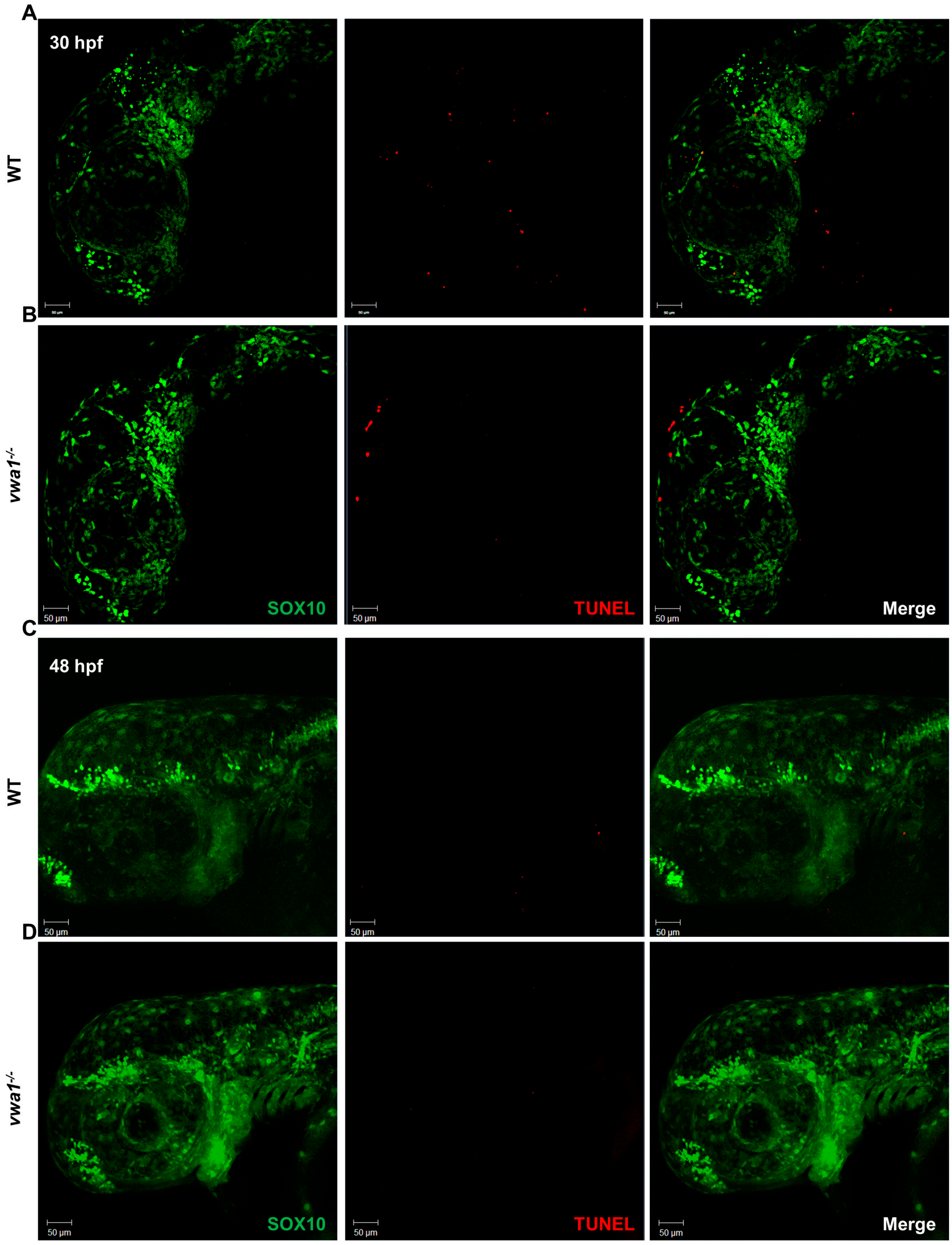

Supplemental Figure S2. Apoptosis did not significantly increase in *vwa1*$^{-/-}$ mutants at 30 and 48 hpf. (**A**, **B**) TUNEL assay (red fluorescence) at 30 hpf demonstrated similar amount of apoptosis cells in the cranial region and dorsal tissues of wildtype controls (WT) and mutants. (**C**, **D**) TUNEL assay (red fluorescence) at 48 hpf showed similar level of apoptosis in WT and mutants
